# Role of HSF1 in cell division, tumorigenesis and therapy: a literature review

**DOI:** 10.1186/s13008-025-00153-1

**Published:** 2025-04-26

**Authors:** Otakar Fiser, Petr Muller

**Affiliations:** 1 Research Centre for Applied Molecular Oncology (RECAMO), Masaryk Memorial Cancer Institute, Brno, Czech Republic; 2 Department of Experimental Biology, Faculty of Science, Masaryk University, Brno, Czech Republic

**Keywords:** Cancer, Cell division, Heat shock, HSF1, HSR, Protein homeostasis

## Abstract

Heat shock factor 1 (HSF1) is the master orchestrator of the heat shock response (HSR), a critical process for maintaining cellular health and protein homeostasis. These effects are achieved through rapid expression of molecular chaperones, the heat shock proteins (HSPs), which ensure correct protein folding, repair, degradation and stabilization of multiprotein complexes. In addition to its role in the HSR, HSF1 influences the cell cycle, including processes such as S phase progression and regulation of the p53 pathway, highlighting its importance in cellular protein synthesis and division. While HSF1 activity offers neuroprotective benefits in neurodegenerative diseases, its proteome-stabilizing function may also reinforce tumorigenic transformation. HSF1 overexpression in many types of cancer reportedly enhances cell growth enables survival, alters metabolism, weakens immune response and promotes angiogenesis or epithelial-mesenchymal transition (EMT) as these cells enter a form of “HSF1 addiction”. Furthermore, the client proteins of HSF1-regulated chaperones, particularly Hsp90, include numerous key players in classical tumorigenic pathways. HSF1 thus presents a promising therapeutic target for cancer treatment, potentially in combination with HSP inhibitors to alleviate typical initiation of HSR upon their use.

## Background

This review aims to provide an accessible yet comprehensive overview of HSF1’s activation, regulation, and functional roles. By integrating the latest research, it seeks to unravel the complexities of HSF1 in cell division and cancer biology, offering a foundational understanding for both curious readers first exploring the topic and seasoned researchers in the field. Emphasis is placed on presenting the subject matter in a clear and engaging manner, ensuring the content serves as a valuable gateway to the expanding body of HSF1 research, while also updating the reader on the most recent discoveries in the field.

## Introduction

The heat shock response (HSR) is a rapid genetic response triggered by a plethora of stresses that disrupt protein homeostasis (proteostasis). The response is primarily mediated by the HSF1 protein, which detects stress and, upon activation, initiates the transcription of genes associated with protein reparation, folding, transportation, complex formation and degradation [1]. HSF1 achieves this by binding to specific DNA sequences known as heat shock elements (HSEs), which are localized within the promoters of genes encoding a superfamily of heat shock proteins (HSPs). These HSPs function as chaperones and co-chaperones, classified by their molecular weight and varying in their effector or complementary roles [2–4]. The stresses that induce HSR are diverse and include heat, oxidative stress, heavy metal and toxin exposure, infection, inflammation, or stress caused by various medications. Basal levels of HSPs provide constant protection to the cell by maintaining protein homeostasis, while the adaptive HSR enhances their expression to manage both acute and chronic stress conditions [5].

Among the six HSF isoforms encoded by the human genome (HSF1, HSF2, HSF4, HSF5, HSFX, and HSFY) [6], HSF1 is regarded as the master regulator of the HSR since the deletion of *Hsf1* leads to insufficient HSR [7, 8]. While HSF2 exhibits limited transactivation activity for HSP expression under stress [9, 10], it seems to hold an important role in the co-regulation of the HSR by both positively and negatively modulating HSF1’s transcriptional activity, for instance, by forming heterotrimers with HSF1 [11]. So far, HSF4 has been reported to have an important developmental function [12]. The remaining HSFs have not yet been extensively studied, however a strong role in gametogenesis is implied [13]. Notably, research indicates that “other mammalian HSFs or distinct physiological pathways do not compensate for HSF1 in the physiological response to heat shock” [14].

Here, we focus on the master regulator of HSR, the HSF1, which trimerizes upon the introduction of stress and translocates to the nucleus, where it initiates transcription of HSE-associated genes. This review aims to provide a concise overview of HSF1’s structure, its mechanism of action, and its many roles in the regulation of cell division and carcinogenesis.

## HSF1 structure, activation and regulation

### Structure of HSF1

**Fig. 1:**
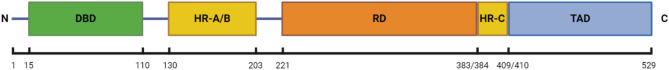
Schematic representation of the domain structure of human heat shock factor 1 (HSF1) [23]. The N-terminal DNA-binding domain enables interaction with heat shock elements (HSEs) in target gene promoters, with binding affinity significantly enhanced upon trimerization. Trimerization is driven by hydrophobic heptad repeats A, B, and C (HR-A/B/C). The transactivation domain (TAD) promotes transcriptional activation of heat shock genes, while its activity is modulated by the regulatory domain (RD)

Under physiological conditions, human HSF1 exists as a monomer with minimal DNA-binding capacity. The monomer comprises several functional domains (Fig. 1) [1]. Upon exposure to stress, HSF1 undergoes trimerization, allowing the N-terminal DNA-binding domain (DBD) to ensure highly specific DNA binding to specific DNA sequences known as heat shock elements (HSEs). The HSEs are characterized by repetitive nGAAn motifs organized in a palindromic arrangement, wherein each nGAAn sequence is followed by its reverse complement nTTCn, resulting in a canonical structure such as nGAAnnTTCnnGAAn. The DBD, characterized by a looped helix-turn-helix structure, recognizes these sequences in the major groove of DNA and ensures robust transcriptional activation and an effective HSR [15, 16].

Trimerization is the pinnacle of HSF1 activation, increasing its binding capacity by several orders of magnitude [16]. This is facilitated by the leucine zipper oligomerization domain, which is connected to the DBD via a flexible linker. It comprises two hydrophobic heptad repeats (HR-A and HR-B). Specific mutations in this region produce a variety of conformational states (e.g. folded monomer, unfolded monomer, stable trimer) [17]. The subsequent regulatory domain (RD) is highly flexible, as it manages the stability and activity of HSF1 through interactions with other domains and undergoes several post-translational modifications that greatly influence the transactivation capacity of HSF1 [18, 19]. The stabilization of the monomeric state and repression of spontaneous trimerization and activity is mediated by yet another hydrophobic heptad repeat (HR-C). This stabilization is thought to be possible through interactions between HR-C and HR-A/B domains, thus suppressing oligomerization. Mutations in HR-C may also result in the formation of constitutively active HSF1 trimers with DNA-binding capability [20]. Notably, the mammalian HSF4 and HSF1 of *Saccharomyces cerevisiae* or *Kluyveromyces lactis* lack the HR-C domain and are intrinsically trimeric [20, 21]. Finally, located at the C-terminal, is the transactivation domain (TAD). It is rich in hydrophobic residues and facilitates appropriate stress response via transcriptional activation of target genes. It is regulated by the RD and conformational changes that lead to trimerization, while it appears to be non-responsive to stress by itself [18, 19, 22].

### Activation and regulation of HSF1

The transition from monomeric to trimeric DNA-binding state is a fundamental aspect of HSF1’s activation across all eukaryotes [9]. This activation initiates HSR, which can be observed microscopically as changes in nuclear morphology. Activated HSF1 localizes to subnuclear structures known as nuclear stress bodies (NSBs) [24]. These NSBs form on specific chromosomal loci, particularly on chromosomes 9, 12, and 15, in response to various stressors, including chaperone or proteasome inhibition [25, 26]. In the context of NSBs, HSF1 binds to Sat III sequences, which, while not canonical HSEs, contain HSE-like motifs due to their AT-rich, repetitive nature. This binding is critical for initiating NSB formation, as it triggers the transcription of non-coding Sat III RNAs, which creates a scaffold for NSB assembly, and recruits necessary transcriptional machinery [27]. Recent studies have shown that the formation of NSBs involves not only HSF1’s DNA binding but also its ability to undergo liquid-liquid phase separation. Zhang *et al.* [28] demonstrated that HSF1 forms small nuclear condensates via liquid-liquid phase separation at heatshockprotein gene loci. This phase separation enriches multiple transcriptional apparatuses through co-phase separation, promoting the transcription of target genes. Additionally, Gaglia *et al.* revealed that HSF1 foci initially form as dynamic, fluid condensates but may transition to a more stable, gel-like state under prolonged stress. This transition impacts the transcriptional activity of HSF1 and the survival of stressed cells, suggesting that phase separation plays a critical role in tuning HSF1’s regulatory functions under proteotoxic conditions [29].

**Fig. 2:**
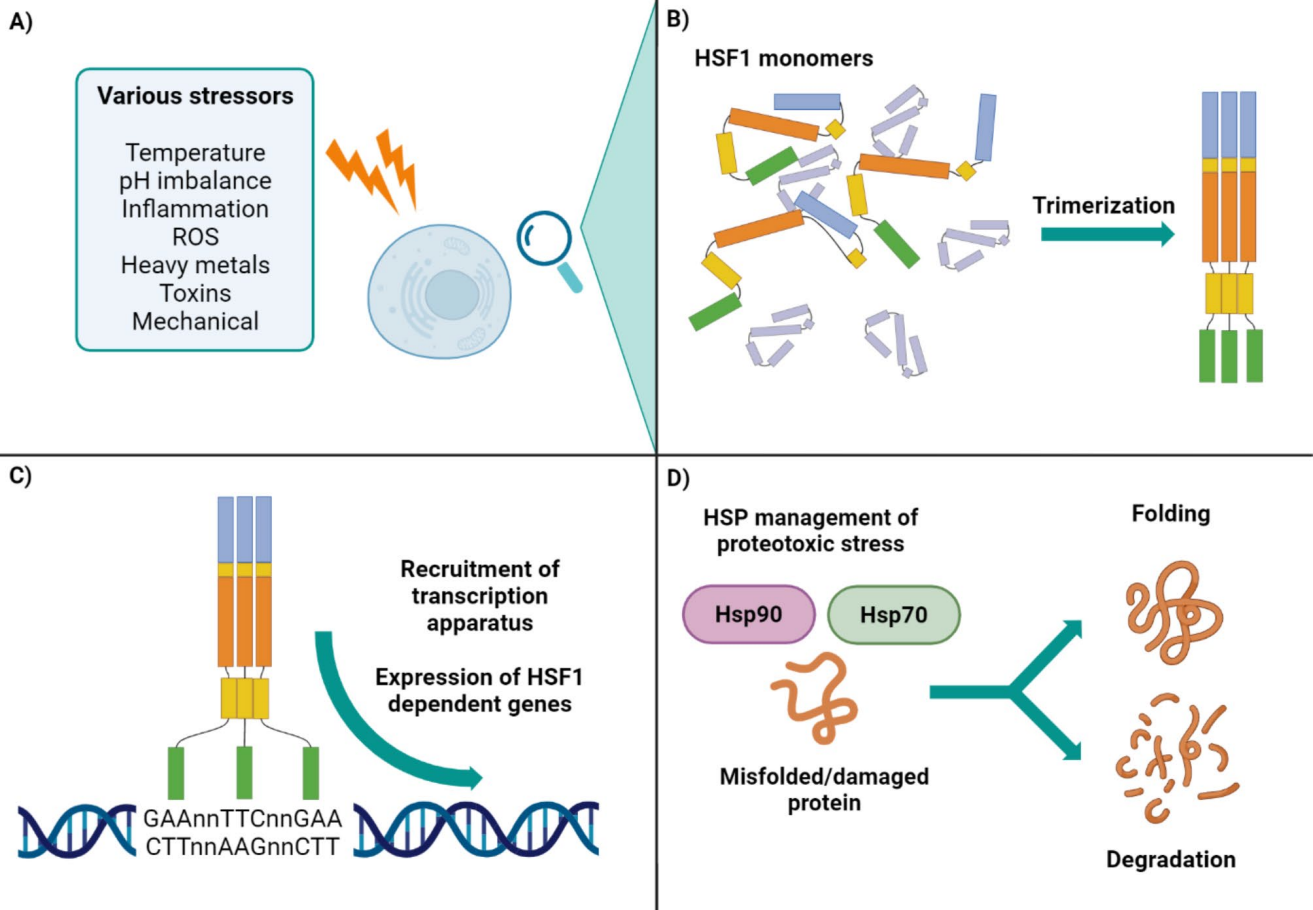
Overview of the heat shock response (HSR), from stress induction to the chaperoning action of heat shock proteins (HSPs). (A) HSR is activated by various stressors, including heat [30], cold [31], acid [32], base [33], inflammation [34, 35], increased reactive oxygen species [36, 37], heavy metals and toxins [38, 39], as well as mechanical stress [40]. (B) Stress triggers the trimerization of HSF1, which leads to its strong nuclear localization and increased DNA-binding capacity [16]. (C) Trimerized HSF1 binds the heat shock elements (HSEs) within target gene promoters, driving the transcription of HSP genes [1]. (D) HSPs, often in collaboration with co-chaperones, mitigate proteotoxic stress through diverse mechanisms, including protein repair, folding, degradation, transport, and complex formation. While a basal level of HSPs supports cellular housekeeping, the HSR significantly upregulates their inducible expression [41]

Despite extensive research, a complete understanding, such as how HSF1 senses stress and how it is regulated, remains elusive, with multiple hypotheses and models suggesting a cooperative role in driving the heat shock response. Although the HSR was originally associated with high temperature induced proteotoxicity [30], it is now well known to be triggered by a diverse array of stressors (Fig. 2a).

It can be hypothesized that HSF1 activation is not entirely dependent on its sensing of stress but perhaps by the recruitment of its regulators. Unsurprisingly, the most widely accepted model of HSF1 activation is the chaperone titration model. It suggests that monomeric HSF1 remains in a multichaperone complex with various HSPs. Upon the introduction of stress and the subsequent increase in protein misfolding, the HSPs dissociate from the complexes enabling the trimerization of HSF1, which ensures transcription of more HSPs. The excess HSPs then help regulate HSF1 activity through a negative feedback loop [42]. It is supposed that increased amounts of Hsp90 and Hsp70 negatively regulate active HSF1 levels, assisting in the attenuation of HSR by inhibiting trimer formation. Additionally, HSF1 transactivation capacity is also modulated by interactions with Hsp70 and Hsp40 [42–44]. Furthermore, trimeric HSF1 has been found to interact with the Hsp90-FKBP52-p23 complex, and inhibition of this complex’s components delays the attenuation of HSF1 DNA-binding activity, suggesting that Hsp90 plays a role in inhibiting trimeric HSF1 [45, 46]. While many studies argue that Hsp90 is the main chaperone regulator, recently Pincus et al. postulated Hsp70 to be the main regulator of yeast HSF1 [47, 48]. This seems to be the case also in *Caenorhabditis elegans* [49] and importantly humans [50]. In this relevant study, Mayer and colleagues describe Hsp70 binding regions at HSF1 in TAD and HR-B, and argue for the main role of Hsp70 in HSF1 regulation. Lastly, Simoncik et al. reported that HSF1 can adopt an unfolded, inactive monomeric conformation in both in vitro and cellular settings without chaperone assistance. This unfolded monomer can undergo a conformational change and assemble into active trimers in response to stress conditions that induce protein denaturation, positioning HSF1 as a direct sensor of proteotoxic stress. Nevertheless, a negative feedback mechanism ensures that once chaperones—upregulated following HSF1 activation—restore cellular proteostasis, they become available to actively disassemble HSF1 trimers and refold HSF1 into its inactive monomeric conformation. This process, primarily mediated by Hsp70, effectively resets HSF1 to its repressed state [51].

While the chaperone titration model is compelling, it is far from being the only mechanism of HSF1 regulation. Direct sensing of, for example, heat, oxidative stress, or low pH levels has been demonstrated in vitro on purified HSF1 and could explain the protein’s ability of rapid activation in cells [36, 52–54]. This intrinsic thermo-sensory ability is further supported by hydrogen-deuterium exchange mass spectrometry (HDX-MS) data, showing significant structural changes in HR-A and HR-C after heat exposure [55]. For some time HSF1 was also considered to be activated via a ribonucleoprotein complex consisting of eEF1a1 and HSR1 (heat shock RNA 1) [56]. The RNA molecule itself was reported to have a thermo-sensing function [57]. However, based on a recent study the concept of eukaryotic heat shock RNA seems invalid [58].

Although HSF1 has been thoroughly investigated in vitro in its purified form and various cell lines, an organismal approach is often missing. Morimoto and colleagues show that the HSR in *Caenorhabditis elegans*, alongside other heat-related processes, is regulated by a thermo-sensory neuron, implying that the HSF1 activation is not necessarily purely cell-dependent, further highlighting the complexity of its regulation [59]. Of note, so far the only known inductor of HSF1 transcription is NRF2 [37].

Evidently, the regulation of HSF1 is influenced mainly by its structure, post-translational modifications, however, are known to tune various steps of its activity such as trimerization, nuclear translocation, DNA binding, transactivation capacity or its half-life [23, 60]. As reported by the PhosphoSitePlus database [61] HSF1 can be modified at 56 residues in humans. Even though many phosphorylations in the RD are considered to be markers of HSF1 activity, Budzýnski et al. demonstrate, that these phosphorylations are not necessary for proper HSF1 cellular localization and DNA-binding capacity [62]. It could be hypothesized that these modifications often merely coincide with fluctuations of HSF1 activity, rather than directly control its state. To fully understand HSF1 regulation, additional research of the exact effects of post-translational modifications is required.

## HSF1 and cell cycle regulation

### HSF1 regulates cell division

Dysregulated cell division is an ever-present phenomenon spanning multiple hallmarks of cancer [63] and over the years, HSF1 has emerged as a critical and multifaceted regulator of mitosis. The connection between HSF proteins and cell cycle regulation was first established in the early 1990s through studies on mutant yeast by Smith and Yaffe. Their research showed that yeast cells carrying a recessive *mas3* mutation—disrupting the gene encoding yeast HSF—experienced cell cycle progression defects [64]. This finding spurred further investigations into HSF1’s role in mitotic division various models, from yeast and mouse embryonic fibroblasts to human cell lines, underscoring its intricate involvement in cell cycle regulation.

Calderwood and colleagues proposed that HSF1 may influence the G1 phase independently of HSP transcription, noting that HeLa cells overexpressing HSF1 exhibited a prolonged G1 phase under stress-free conditions [65]. Later, He and Fox provided additional insights, demonstrating that while HSF1 expression levels remain stable throughout the cell cycle, its DNA-binding activity fluctuates. HSF1 binding to HSEs doubled during the S phase compared to G1 and G2/M phases, a finding later supported by Gross and colleagues [66, 67].

An unexpected interaction, described by Lee’s team, reported that HSF1 interacts with CDC20 during cell division, indirectly inhibiting APC/C activity. In early mitosis, HSF1 is phosphorylated by polo-like kinase 1 (Plk1) at serine 216. This phosphorylation allows for the binding of CDC20 (disrupting the coupling of CDC20 and CDC27, canonical subunits of the APC/C complex) [68, 69]. This modification is necessary for HSF1 degradation through the SCF^β-TrCP^ pathway at the spindle pole, facilitating APC/C activation and mitotic progression [70, 71]. These findings suggest that HSF1 degradation via its interaction with CDC20 is crucial for completing mitosis and maintaining cell cycle control.

### HSF1 and stress during mitosis

As cell cycle progresses, the accessibility to DNA for transcription to occur is variably limited, because the chromatin’s structure folds and unfolds across different phases. During mitosis, when chromatin is highly condensed, most transcription factors are unable to bind effectively, leaving dividing cells particularly vulnerable to stress [72]. HSF1’s chromatin access is markedly reduced during mitosis, from 1,242 binding sites in heat-stressed cycling cells to just 35 sites in mitotic heat-stressed cells. The few retained HSF1 loci primarily encode HSPs, such as Hsp90. Interestingly, HSF2 maintains hundreds of binding loci across both cycling and mitotic cells, though these interactions are likely insufficient to drive transcription due to RNA Polymerase II inactivity during mitosis [73, 74].

Newly synthesized proteins are particularly sensitive to proteotoxic stress, which may serve as the primary trigger for HSF1 activation, as suggested by Tye and Churchman [75]. Various stressors capable of inducing HSR can inhibit cell cycle progression by affecting its checkpoints. Given this, proliferating cells predictably exhibit high HSF1 activity during the S phase. HSPs– the downstream effectors of HSF1– hold an important role in the regulation of proteins relevant for mitotic progression. For example, Hsp90 interacts closely with CDK1 in fission yeast, underscoring its regulatory role [76–78]. Sawarkar and colleagues further identified Hsp90 as essential for maintaining HCFC1-associated cell cycle genes, suggesting that HSPs are crucial for modulating cell cycle arrest and re-entry under stress conditions [79]. Additionally, evidence supports an interplay between HSF1 and p53. Logan and colleagues demonstrated that HSF1 enhances p53-mediated transcription, as silencing either HSF1 or p53 significantly reduced the expression of key p53 target genes, including p21 and PUMA, which are pivotal for cellular stress responses [80]. Li and Martinez further highlighted the importance of HSF1 for p53 nuclear localization and checkpoint activation [81].

### HSF1’s effects on cell division in cancer

The established links between HSF1 and cell cycle regulation strongly indicate its role in cancer progression. Wu and colleagues observed elevated HSF1 mRNA and protein levels in metastatic prostate cancer cell lines (PC3M) compared to non-metastatic lines (PC3). This increase also extended to key HSF1 downstream targets, including Hsp27, Hsp70, and Hsp90. Notably, in patient-matched samples, HSF1 expression was lower in normal cells than in cancerous tissues, highlighting this differential expression pattern [82]. In concordance with the importance and increased expressions of HSF1 in cancer, the silencing of HSF1 led to a significant decrease in the proliferative capability in human melanoma cells [83]. Consistent with these findings, Calderwood’s work introduced a dominant-negative HSF1 (DN-HSF1) construct to inhibit HSF1 transcriptional activity in prostate carcinoma cells. This intervention reduced aneuploid cell populations and promoted cyclin B1 degradation, a process essential for completing mitosis [84]. A surprising finding highlighting the complex nature of HSF1 in cell division regulation came into play, when Momonaka *et al.* described a significantly reduced proliferation in HeLa cells expressing constitutively active HSF1 [85].

Evidently, both HSF1 knockdown and overexpression contribute to cell cycle instability. Overexpression of HSF1 has been associated with an increased proportion of cells in the G1 phase, which may lead us to a conclusion that HSF1 could potentially play a role in the suppression of cancer growth, however, more probably points to a more complicated role in cell cycle regulation [65, 85]. Cancer cells often endure genomic instability and fluctuating microenvironments, conditions where elevated HSF1 activity, based on its reparative functions, likely support survival. The role of Hsp90 and the inefficiency of HSR compensation during division provide a rationale for targeting Hsp90 in highly proliferative and metabolically active cancers. Several clinical trials testing Hsp90 ATPase inhibitors have demonstrated promising anti-cancer effects, underscoring the need to consider the specific environmental context of each tumor [86, 87].

### Conclusions on HSF1’s role in cell division

In summary, the available data highlights HSF1’s crucial role in cell division, particularly during key checkpoints where cells determine whether to proceed through the cycle. Its diverse functions and complex interactions across all phases present significant challenges for cell cycle and HSF1 researchers alike. Moreover, distinguishing between the effects of HSF1 in stressed versus non-stressed conditions remains essential. Given its rapid activation in response to stress, meticulous handling of samples is necessary to obtain reliable data. For instance, trypsinization—the most common method for subculturing cells—induces significant changes in the proteome and alters levels of HSF1 downstream targets such as Hsp60 [88].

## HSF1 promotes cancer development

**Fig. 3:**
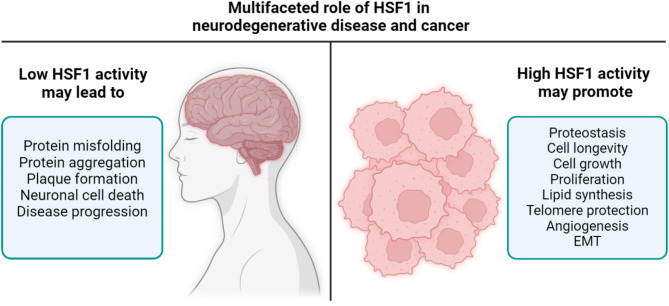
Multifaceted role of human heat shock factor 1 (HSF1) in neurodegenerative disease and cancer

Properly functioning HSF1 and an intact HSR provide many benefits to neural cells, notably through their anti-proteotoxic effects, which are crucial for protecting against aging and age-related pathologies. While impaired HSF1 activation does not directly cause neurodegenerative diseases, it contributes to plaque formation, neuronal cell death, and disease progression due to increased protein misfolding and aggregation [6]. Contrary to its helpful role in the central nervous system, HSF1 has been observed to quite effectively enable tumorigenic growth (Fig. 3). Although HSF1 is not considered to be a tumor suppressor or a typical oncogene, it influences signaling pathways associated with oncogenic hallmarks including growth, proliferation, apoptosis, metabolism, angiogenesis or cell motility [89]. Numerous in vitro studies have demonstrated that cancer cell lines are highly dependent on HSF1, with significantly reduced growth rates observed in HSF1-depleted hepatocellular carcinoma (HCC), melanoma, multiple myeloma, malignant peripheral nerve sheath tumors, and in breast and pancreato-biliary cancers [90–95]. In contrast, non-cancerous cell lines show little to no effect from HSF1 deletion [92, 94]. Neurodegenerative diseases are often characterized by the accumulation of misfolded proteins and an age-related decline in the neuronal capacity to counter proteotoxic stress. HSF1 activation has been shown to protect neurons from apoptosis and cell death through its chaperone activity [96]. Pharmacological enhancement of HSF1 activity could offer therapeutic and preventive benefits for individuals at risk of or suffering from neurodegenerative diseases. However, overexpression of HSF1 may promote tumorigenesis, creating a state of “HSF1 addiction” in cancer cells. Thus, controlled, chronic activation of HSF1 presents a potential therapeutic strategy for neurodegenerative conditions [97].

In concordance with *in vitro* studies;*in vivo* and clinical studies also profoundly support strong pro-oncogenic function of HSF1. In many human cancers including HCC, breast cancer, endometrial carcinoma, oral squamous cell carcinoma or prostate cancer; increased HSF1 levels compared to non-cancerous tissues are observed. The increased HSF1 concentrations are associated with poor prognosis, larger tumor size and shorter overall and disease-free survival [98]. HSF1 mRNA levels are increased in various cancers, including breast, endometrial, and ovarian tumors [99–101], often due to *HSF1* gene amplification and mutations in splicing factors [101, 102]. Additionally, in 2007 it was noted that HSF1 contributed to lymphoma development in *p53-/-* mice, suggesting a role in lymphomagenesis [103]. Further research by Dai’s team highlighted HSF1’s protective role in tumors of *p53* and *Ras* mutated mice [94]. In a different study the authors proposed a mechanism by which HSF1 promotes the growth of pre-malignant cells and HCC by stimulating lipid synthesis and cellular longevity in the presence of carcinogens, with *HSF1-*deficient mice showing reduced cancer progression [104]. HSF1’s prooncogenic role has also been described in mouse mammary tumors. The deletion of *HSF1* reduced tumorigenesis and metastasis in *ERBB2* overexpressing cells, decreasing tumor growth rate and suppressing angiogenesis [105, 106].

Support for the role of HSF1 in cancer has been further reinforced by various independent studies using xenograft models. In these, *HSF1* gene knockdowns resulted in impaired growth rates, reduced invasion and metastatic capabilities of xenografted HCC and melanoma cells in immunocompromised mice [107, 108]. Overexpression of HSF1, on the other hand, exacerbated pro-invasive and migration capabilities of melanoma xenografts in vivo [109, 110].

Overall, it is apparent that HSF1 activity contributes to malignant transformation and supports tumor progression. However, the question remains whether elevated HSF1 activity in tumors is solely a response to proteotoxic stress associated with cancer or if its expression and activation are directly regulated by oncogenic signaling.

## Molecular dynamics of HSF1 in oncogenesis

As previously discussed, HSF1 is typically activated by an array of stressors. Cancer cells, however, appear to keep HSF1 constitutively activated [94, 111], indicating a state of “HSF1 addiction” driven by continuous cellular stress. Factors such as acidic or hypoxic microenvironments, elevated protein synthesis, aneuploidy, genetic mutations, and metabolic stress contribute to this persistent proteome stress. Despite this understanding, the complete picture of continual HSF1 activation remains elusive. The HSF1 protein is, for example, extensively phosphorylated. These modifications act as both stimulatory and inhibitory factors and have been well mapped out, albeit their complex interactions in HSF1 regulation must be further studied [112]. Various signaling pathways including MAPK/ERK, PI3K/Akt, LKB1/AMPK, GSK-3, JNK, p38/MAPK, PKC, PKA, PLK1, CK2, DYRK2, IER5, and Sirtuin 1 regulate HSF1 activation through phosphorylation, dephosphorylation, or deacetylation [60, 113–115] only adding layers of complexity to the difficult research of HSF1 regulation.

**Fig. 4:**
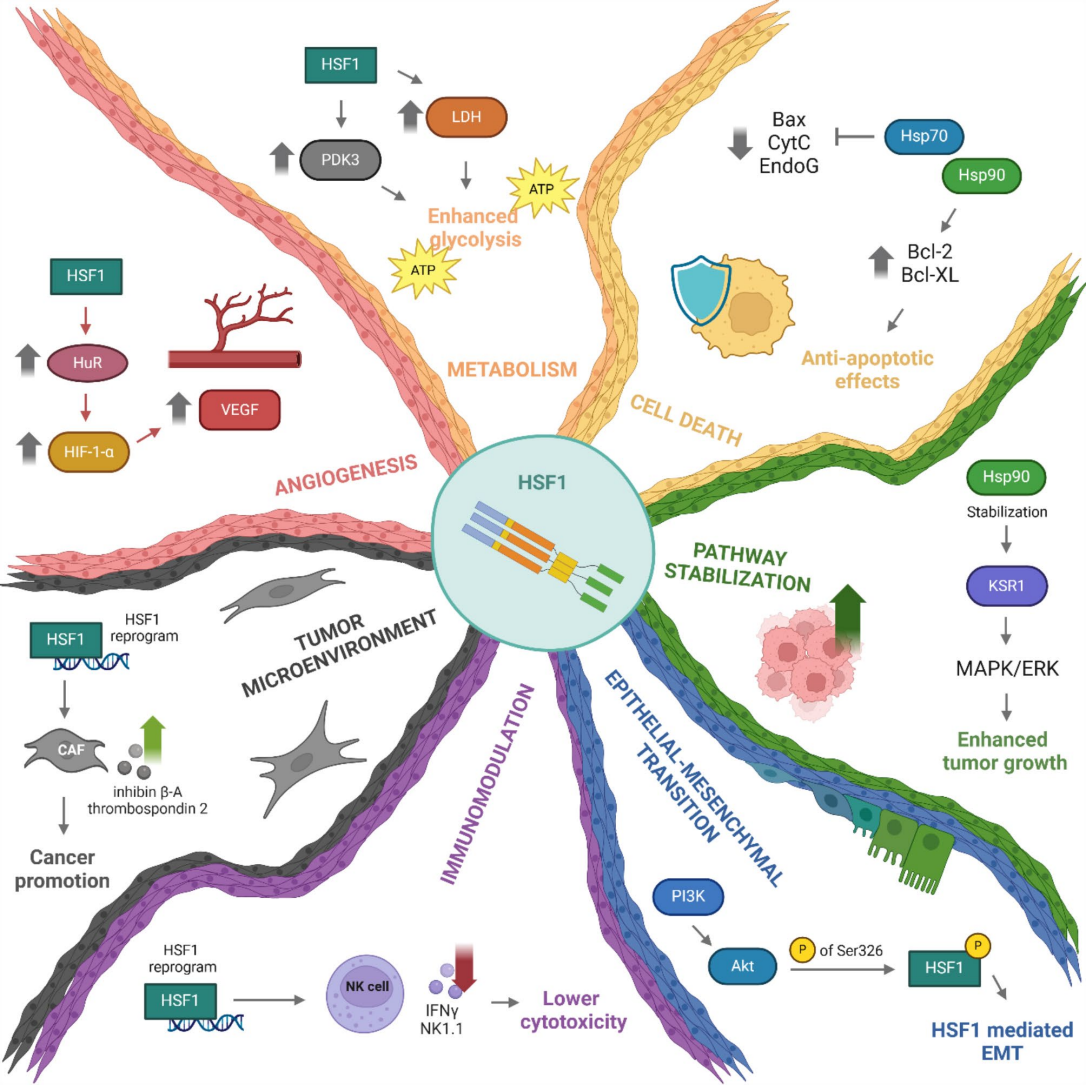
The many effects of HSF1 in cancer. HSF1 plays diverse roles in tumorigenesis by modulating various cellular processes. It drives metabolic reprogramming in cancer cells, aiding the shift to aerobic glycolysis [116, 117]. HSF1 also promotes tumor survival by preventing cell death by altering the levels of various pro- and anti-apoptotic proteins [118–121]. It is also known for indirectly stabilizing oncogenic signaling pathways– for example, the MAPK/ERK cascade, which regulates KSR1, a key scaffolding protein required for proper pathway activation [92]. Additionally, phosphorylation of HSF1 at serine 326 is associated with epithelial-to-mesenchymal transition (EMT), and enhanced tumor invasiveness [113]. Beyond cancer cells, HSF1 can influence the tumor microenvironment (TME) by reprogramming natural killer (NK) cells, leading to reduced cytotoxicity against tumor cells [122]. Furthermore, HSF1 expression in cancer-associated fibroblasts (CAFs) contributes to tumor progression through the secretion of extracellular vesicles (EVs) containing pro-tumorigenic factors such as inhibin β-A and thrombospondin 2 [123]. These diverse functions establish HSF1 as a central and complex regulator of cancer progression

### HSF1 facilitates pathway stabilization

To understand how HSF1 activation influences cellular behavior, it is essential to study the effectors of HSR, particularly the inducible chaperones Hsp70 and Hsp90, which are among the most abundant. The vast range of HSP targets suggests that the consequences of their overexpression are very wide and non-specific. Hsp70 and Hsp60 seem to interact with all conformationally unstable proteins [124]. Hsp90 supports the stability and function of hundreds of client proteins including mainly kinases (60%), 3 ubiquitin ligases (31%) ad transcription factors (< 7%) [125].A list of the entire interactome is being maintained and updated by Didier Picard [126]. Many of these clients are involved in pathways associated with oncogenic progression (e.g. ErbB2, Bcr-Abl, VEGFR, Akt, Met, p53). For instance, KSR1 (kinase suppressor of RAS 1) is a known client of Hsp90, meaning Hsp90 chaperones KSR1 to prevent its degradation and maintain its function. In this way HSF1 has been shown to support MAPK signaling by stabilizing KSR1, a scaffolding protein critical for MAPK/ERK pathway activation (Fig. 4) [92]. Comparably, EGFR, Akt and MIF pathways, all important in tumorigenesis, can be disrupted by an *HSF1* knockdown [127–129].

### HSF1 enables epithelial-mesenchymal transition

HSF1’s role in oncogenesis extends beyond pathway stabilization. In ovarian cancer cells, HSF1 deficiency impairs the expression of key epithelial-mesenchymal transition (EMT) genes, including SLUG, SNAIL, ZEB1, and TWIST1 [130]. In another study it was observed that PI3K, through Akt, modulates HSF1 by phosphorylation of serine 326, linking PI3K signaling to HSF1-mediated EMT in HER2-positive breast cancer cells (Fig. 4) [113]. Furthermore, the deletion of *HSF1* significantly reduced EMT of mammary epithelial cells in transgenic mice [106].

### HSF1 plays a role in regulation of cell death

The ability of cells to undergo apoptosis is a crucial prerequisite in the organism’s protection against cancer. Many types of therapies depend on initiation of programmed cell death induced by their effects, however resistance to apoptosis due to alterations in its pathways is a common phenomenon [131]. Whether the cell enters into apoptosis is often in the hands of the balance between pro-apoptotic and anti-apoptotic proteins. HSPs often play a role in this delicate balance. Research on Hsp70 points to solid anti apoptotic activity. It has been shown to inhibits activation of the pro-apoptotic Bax [118]; prevent downregulation on anti-apoptotic MCL-1 [118]; interfere with cytochrome C [119]; or inhibit endonuclease G, thus reversing DNA fragmentation [120]. Supporting this, Hsp70 member 6 (HSPA6) physically interacts with anti-apoptotic Bcl-XL and increases its levels [132]. Furthermore, HSF1 expression leads to an upregulation of BAG3 (Bcl-2-associated athanogene-3)– a Hsp70 cochaperone– which appears to play a role in the stabilization of Bcl-2 family proteins, promoting apoptosis evasion [133]. This HSF1/Hsp70/BAG3 axis is associated with fortified cell resistance to treatment in glioma and gastric cancer [134, 135]. In similar fashion, Hsp90 exerts a protective effect on cancer cells via elevation of Bcl-2 and Bcl-XL expression (Fig. 4), or also by attenuation of cleaved caspase-3 expression, achieved by the downregulation of TLR-4 and ErbB2 receptors [121]. Furthermore, a direct association between Hsp60 and cyclophilin D, a component of the mitochondrial permeability transition pore, has been observed. Silencing of Hsp60 led to caspase dependent apoptosis and growth inhibition of intracranial glioblastoma [136]. These examples show the significant roles HSPs– and therefore HSF1– can play in the evasion of cell death via pathway stabilization.

On the other hand, some studies also point to the ambivalent nature of HSF1 in its pro-apoptotic and anti-apoptotic effects. For example, Benderska et al. describe a novel observation of the typically pro-survival HSF1 being redirected by TNF to a pro-apoptotic program [137]. In concordance with this surprising revelation, HSF1 has been observed to bind sequences in the introns of the *NOXA* gene, upregulating its expression and thus promoting apoptosis in heat sensitive cells [138]. Despite the evident anti-apoptotic effects of HSF1 and its downstream effectors– the HSPs–mechanisms underlying the relationship between HSF1 and apoptosis still need to be further elucidated.

### HSF1 influences metabolism in cancer

Cancer cells are known to undergo a metabolic shift from the preferential oxidative phosphorylation to aerobic glycolysis referred to as the „Warburg effect“ [139] and HSF1 is not exempt from playing a role in this phenomenon. HCC and breast cancer have been shown to depend on HSF1 to sufficiently express lactate dehydrogenase (LDH), where LDH is crucial for glycolytic efficiency and further malignant growth and promotion. Down-regulating HSF1 leads to decreased LDH levels and therefore ineffective glycolysis, halting growth of cancer cells [117]. Similarly, HSF1 promotes expression of pyruvate dehydrogenase kinase 3 (PDK3), which enhances glycolysis and analogically supports cancer progression and resistance (Fig. 4). A positive feedback loop is implemented, as PDK3 prevents HSF1 from degradation via FBXW7-dependent polyubiquitination [116].

HSF1 is further incorporated in cancer metabolism by positively regulating biosynthesis of mevalonate and cholesterol. These molecules are crucial for the RAS-MAPK pathway [140]. Accordingly, mouse T-cell acute leukemia (T-ALL) displayed a dependency on HSF1 to help maintain MAPK/ERK signaling, as well as ATP-producing capacity. Here, HSF1 depletion leads to an energy saving state stemming from reduction of mTORC1 activity, effectively slowing down growth and reducing oncogenic signaling [141]. HSF1 was also found to promote malignancy by suppressing AMPK, therefore reprogramming lipid metabolism and enhancing protein lipidation [142].

### HSF1 indirectly influences angiogenesis

Notably, HSF1 positively regulates human antigen R (HuR) transcription, which is essential for VEGF pathways involved in hypoxia-induced angiogenesis [143]. HSF1 deletion leads to a downregulation of HuR resulting in impaired HIF-1-α translation, thereby hindering tumor angiogenesis (Fig. 4) [105]. An mTORC2/Akt/HSF1/HuR feed-forward loop, promoting Rictor via HSF1-induced HuR activity, is furthermore associated with increased growth rates and aggressiveness in glioblastoma [144].

### HSF1 alters the tumor microenvironment

Given HSF1’s truly multifaceted role, it comes as no surprise that it also influences tumor microenvironment (TME)– a space consisting of various cell types, extracellular vesicles (EVs) and signal molecules. Cancer-associated fibroblasts (CAFs) are considered to be significant components of the TME as they assist in angiogenesis, invasiveness or resistance, among others [145]. HSF1 has been reported to reprogram CAFs leading to expression of TGF-β and SDF1, supporting malignancy. Additionally, such high stromal HSF1 activity strongly correlated with poor prognosis in early-stage breast and lung cancer [146]. Significant correlation between CAF HSF1 expression and poor prognosis and overall survival was observed in oral and esophageal squamous carcinoma [147, 148]. We come closer to a mechanistic understanding of HSF1’s effect on CAFs with a study by Grunberg et al., where HSF1 reportedly upregulated the synthesis and EV secretion of inhibin β-A and thrombospondin 2 promoting gastric cancer (Fig. 4) [123].

HSF1’s effects on its vicinity are further reinforced by HSP export into the extracellular space via exosomes, likely independent of the classical secretory pathway or lipid raft-dependent mechanisms [149]. Through this transmission, HSPs may extend their stabilizing effects on proteins within neighboring cells, supporting a broader malignancy-promoting network by stress rescue of tumor cells [150].

### HSF1 linked Immunomodulation in TME

Recently, in numerous studies extracellular HSPs exhibited an immunomodulating function leading to interactions with macrophages, NK cells, T-lymphocytes, B-lymphocytes, dendritic cells in TME [151]. However, direct HSF1 effect on immunomodulation is also present. Research published in Nature Cell Biology revealed that in TME, activation of HSF1 in NK cells leads to a decrease in the expression of effector molecules, such as NK1.1 and IFNγ, thereby impairing their cytotoxic function against tumor cells (Fig. 4) [122]. Additionally, HSF1 seems to prevent CD8 + T-cell recruitment to breast cancer microenvironment by downregulating the expression of CCL5. This significantly hampers immune response to cancer cells in the tissue. Knockdown of HSF1 in breast cancer cells led to decreased tumor size and increased CD8 + T cell infiltration, which was mediated by CCL5 [152].

### HSF1 plays a surprising role in tumor amyloidogenesis

A particularly intriguing finding by Dai and colleagues links HSF1 pathway disruption to amyloidogenesis in tumor cells [153]. While amyloid plaque formation is primarily known to exacerbate neurodegenerative disease, their research suggests that amyloidogenesis may play a tumor-suppressive role, hampering melanoma growth and invasiveness in vivo and potentially offering a novel therapeutic approach [153].

### HSF1 holds a multifaceted role in cancer

In summary, HSF1 clearly acts as a truly multifaceted player in cancer promotion. It holds roles in tumor growth, survival promotion, EMT, metabolism alteration, immunomodulation, and angiogenesis. It also likely enables cancer cell viability via the chaperoning of innumerable client proteins spanning molecules integral for well-described cancer pathways, and thus aids in oncogenic progression. While HSF1 disruption can reduce tumor growth, it evidently may also induce amyloid formation, potentially providing a tumor-suppressive effect. Additionally, HSF1’s influence extends to cells of the TME by reprograming the stroma and influencing the invasion of immune elements, while indirectly facilitating the exportation of HSPs via exosomes, further emphasizing its significance in cancer biology.

## HSF1 as a viable option in therapy

HSF1 is inherently challenging to target due to its molecular structure and complex regulation. This has directed research efforts toward inhibiting pathways that enable HSF1 activation, rather than directly targeting the protein itself. As previously discussed, HSF1 is overexpressed in various cancers and plays a key role in tumorigenesis. It is also linked to increased chemotherapy resistance [154]. Despite its importance in cancer biology, efforts to target HSF1 therapeutically remain in preclinical stages, with most approaches facing significant limitations.

One potential strategy for HSF1 suppression involves RNA interference therapeutics, although this area remains largely unexplored [60, 155]. The primary approach to date has focused on small-molecule inhibitors, which often lack specificity and potency and target HSF1 indirectly. However, some compounds displayed tumor growth limitation with relatively low toxicity to non-cancer tissue in animal studies. A notable direct inhibitor of HSF1, DTHIB (Direct Targeted HSF1 InhiBitor), achieves its effect by accelerating nuclear HSF1 degradation and has shown strong efficacy in multiple models of resistant prostate cancer [156]. Optimistically, the indirect inhibitor CCT361814 (NXP800), discovered through phenotypic screening, has advanced to Phase I clinical trials, with completion expected by December 2025 [157–159]. Notably, subsequent research on NXP800 provided a deeper understanding of its mechanism of action, revealing that it functions as an activator of the General Control Nonderepressible 2 (GCN2) kinase. Activation of GCN2 leads to phosphorylation of the eukaryotic translation initiation factor 2 (eIF2), resulting in reduced overall protein synthesis and the induction of stress-adaptive genes like *ATF4*, ultimately causing cancer cell death [160].

Indirect targeting of HSF1 through components of the protein homeostasis system, such as Hsp90 and the proteasome, has seen more clinical progress. Tumor cells are particularly sensitive to these inhibitors, likely due to elevated proliferative activity, protein synthesis, and proteotoxic stress [161]. However, compensatory activation of HSF1 tends to occur, leading to induction of the HSR and overproduction of other HSPs [161, 162]. These findings suggest that combining such inhibitors with HSF1-targeted therapies could enhance their effectiveness.

Given the critical role of HSF1 in tumorigenesis and drug resistance, targeting this pathway offers immense therapeutic potential. Combining indirect HSF1 inhibitors such as NXP800 with inhibitors of protein homeostasis pathways or standard cancer treatments could improve outcomes and help overcome resistance mechanisms. Future research and clinical trials will likely focus on these synergistic strategies to maximize the therapeutic impact of HSF1 inhibition.

**Conclusions**.

In summary, HSF1 is a key regulator of cellular homeostasis, orchestrating the HSR to maintain protein integrity under stress. Its multifaceted roles extend beyond stress adaptation to include regulation of cell division and involvement in both neuroprotection and tumorigenesis. The research on HSF1 is proving to be a demanding endeavor given its obvious role in both housekeeping functions and inducible necessities to facilitate an adequate response to proteotoxic stress. While HSF1 offers protective effects in neurodegenerative diseases, its overexpression in cancer promotes tumor growth, highlighting its paradoxical nature. Due to its central role in the stabilization of tumorigenic pathways leading to an enhanced capability for cells to exhibit the many hallmarks of cancer, HSF1 presents a promising therapeutic target. However, its complex structure, activation and regulation make targeting HSF1 with small nuclear inhibitors an uneasy task. Still, further research into the precise mechanisms of HSF1 activation and its interactions will be essential for developing targeted strategies to modulate its activity in disease contexts. Due to these problems, heightened attention should also be given to advancements on the frontier of RNA interference therapies, though they too still face their own challenges in maximizing therapeutic potential.

## Data Availability

No datasets were generated or analysed during the current study.

## Abbreviations

AMPK: AMP-activated protein kinase
APC/C: Anaphase-promoting complex/cyclosome
ATF4: Activating transcription factor 4
β-TrCP: β-transducin repeat-containing protein
CDC: Cell division cycle protein
Cdc2: Cyclin-dependent protein kinase Cdk1
CDK: Cyclin dependent kinase
CK2: Casein kinase 2
DBD: DNA binding domain
eEF1a1: Elongation factor 1-alpha 1
EGFR: Epidermal growth factor
EMT: Epithelial-mesenchymal transition
FKBP52: FK506-binding protein 4
GCN2: General control nonderepressible 2
GSK-3: Glycogen synthase kinase 3
HCC: Hepatocellular carcinoma
HCFC1: Host cell factor 1
HDX-MS: Hydrogen-deuterium exchange mass spectrometry
HIF-1-α: Hypoxia-inducible factor 1-alpha
HR-A/B or C: Hydrophobic heptad repeat A/B or C
HSE: Heat shock element
HSF: Heat shock factor
HSP: Heat shock protein
HSR: Heat shock response
HSR1: Heat shock RNA 1
HuR: Human antigen R
IER5: Immediate early response gene 5 protein
IFNγ: Interferon gamma
JNK: cJ-un N-terminal kinase
KSR1: Kinase suppressor of RAS1
LKB1: Liver kinase B1
MAPK: Mitogen-activated protein kinase
mas3: Gene for heat shock factor in yeast
MIF: Macrophage migration inhibitory factor
NRF2: Nuclear factor erythroid-derived 2-like 2
NSB: Nuclear stress body
NK1.1: NK cell marker
p23: Hsp90 co-chaperone
PKA: Protein kinase A
Plk1: Polo-like kinase 1
PUMA: p53 upregulated modulator of apoptosis
RD: Regulatory domain
SCF: Skp, Cullin, F-box containing complex
SDF1: Stromal cell-derived factor 1
TAD: Transactivation domain
TGFβ: Transforming growth factorβ
VEGF: Vascular endothelial growth factor
VEGFR: Vascular endothelial growth factor receptor
